# Build-UPS and break-downs: metabolism impacts on proteostasis and aging

**DOI:** 10.1038/s41418-020-00682-y

**Published:** 2021-01-04

**Authors:** Franziska Ottens, André Franz, Thorsten Hoppe

**Affiliations:** 1 Institute for Genetics and Cologne Excellence Cluster on Cellular Stress Responses in Aging-Associated Diseases (CECAD), University of Cologne, Cologne, Germany; 2 Center for Molecular Medicine Cologne (CMMC), University of Cologne, Cologne, Germany

**Keywords:** Protein quality control, Metabolic pathways, Ageing

## Abstract

Perturbation of metabolism elicits cellular stress which profoundly modulates the cellular proteome and thus protein homeostasis (proteostasis). Consequently, changes in the cellular proteome due to metabolic shift require adaptive mechanisms by molecular protein quality control. The mechanisms vitally controlling proteostasis embrace the entire life cycle of a protein involving translational control at the ribosome, chaperone-assisted native folding, and subcellular sorting as well as proteolysis by the proteasome or autophagy. While metabolic imbalance and proteostasis decline have been recognized as hallmarks of aging and age-associated diseases, both processes are largely considered independently. Here, we delineate how proteome stability is governed by insulin/IGF1 signaling (IIS), mechanistic target of Rapamycin (TOR), 5′ adenosine monophosphate-activated protein kinase (AMPK), and NAD-dependent deacetylases (Sir2-like proteins known as sirtuins). This comprehensive overview is emphasizing the regulatory interconnection between central metabolic pathways and proteostasis, indicating the relevance of shared signaling nodes as targets for future therapeutic interventions.

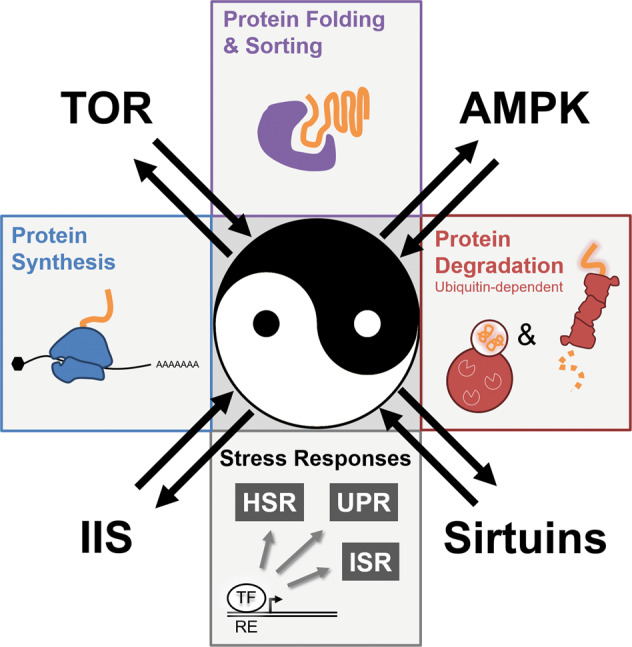

## Facts

- Metabolic imbalance and proteostasis decline have been recognized as hallmarks of aging and age-associated diseases, both processes are largely considered independently.
- Coordination of protein and metabolic homeostasis is crucial for balanced energy homeostasis, organismal physiology, and health.
- Both, metabolic imbalance and proteostasis decline are causally linked to aging and aging-associated pathologic disorders.

## Open questions

- Common signaling nodes and shared mechanisms of proteostasis networks and metabolism remain to be identified.
- How are metabolic perturbations and proteotoxicity interconnected and are protein quality control mechanisms relevant targets for clinical intervention?
- How do changes in the activity of growth pathways that significantly shape the cellular proteome, affect organismal viability, and physiology?
- How are environmental and/or nutritional cues incorporated into proteostasis pathways and could these cues be used as health benefit?

## Introduction

### Proteostasis surveillance mechanisms

The composition and functional integrity of the cellular proteome is under constant surveillance to maintain a balanced state of proteostasis. The importance of a stable proteome for physiological health is underscored by numerous human pathologies that are causally associated with proteostasis impairment. Neurodegenerative diseases such as Parkinson’s-, Huntington’s-, and Alzheimer’s disease (PD, HD, AD), or amyothrophic lateral sclerosis (ALS) are characterized by prototypic deposition of insoluble protein aggregates. Cancer cells also display hallmarks of defective proteostasis, which has led to the discovery of chemotherapeutic interventions to selectively eliminate tumors that are consequently vulnerable to proteotoxic treatment [1]. Thus, proteostasis imbalance has become recognized as a central driving force and hallmark for age-associated pathologies [2].

**Fig. 1:**
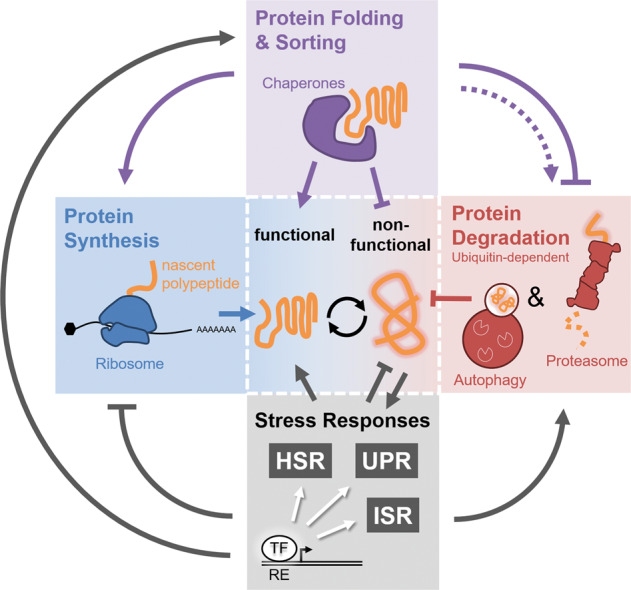
Proteostasis pathways. Schematic overview of cellular proteostasis pathways. Protein (orange) synthesis at the ribosome (blue) is controlled at multiple level through regulated translation initiation or elongation. Already during translation molecular chaperones facilitate native protein folding and compartment-specific sorting (purple). Cytosolic and compartment-specific chaperones play a crucial role in protein quality control by supporting functional folding or prevention of protein aggregation (purple). In addition, dedicated molecular chaperones also facilitate protein ubiquitylation and proteasomal targeting or promote aggregate formation and autophagic disposal (dashed purple arrow). In case proteins are terminally misfolded or not used, proteolysis by the proteasome and autophagosome catalyze turnover (red). Noteworthy, both proteasomal and autophagosomal protein degradation are triggered by substrate ubiquitylation. Dedicated stress-response pathways coordinate proteostasis mechanisms (gray). Proteotoxic stress results in activation of specific transcription factors (TF) that bind to distinct regulatory elements (RE) in the DNA and mediate stress-compensatory responses to restore proteostasis. See text for details.

Eukaryotic cells exhibit different protein quality control mechanisms and surveillance strategies to counteract ultimate proteostasis collapse (Fig. 1). The abundance of proteins and the composition of the cellular proteome are largely regulated at the level of protein biosynthesis. Regulation of protein translation is coupled to mRNA processing and is further controlled at different steps, including initiation, elongation, as well as termination [3]. Molecular chaperones facilitate functional folding of nascent polypeptides and assist in correct subcellular localization of matured proteins [4]. Otherwise, the ribosomal-quality control pathway eliminates aggregation-prone translation products as a consequence of impaired translation fidelity [5]. Following translation, cytosolic and compartment-specific chaperoning systems facilitate protein transport across membranes as well as supervise the integrity of the proteome either by assisting in functional (re-)folding or targeted protein turnover [6, 7]. In case proteins are permanently damaged and cannot be restored by chaperoning systems, protein degradation mechanisms catalyze proteolytic disposal. In this context, the ubiquitin–proteasome system (UPS) and macro-autophagy (hereafter autophagy) constitute the two major proteolytic pathways in eukaryotic cells [8]. Protein degradation by the 26S proteasome is triggered by conjugation of ubiquitin to substrate proteins [9]. The ubiquitylation reaction involves an enzymatic cascade of so called E1, E2, and E3 enzymes, earmarking proteins for shuttling to and proteolysis by the proteasome. In contrast, autophagic degradation of proteins and macromolecules is triggered by engulfment of cytosolic content by de novo formation of a double-layered membrane, the phagophore, which upon maturation to a vesicular autophagosome fuses with the lysosome to allow degradation of its content by lysosomal hydrolases [10]. The content of the phagophore/autophagosome is largely dependent on autophagy receptors that mediate selective engulfment of cargo substrates. Dedicated receptors mediate autophagic degradation of ubiquitin conjugates by recruiting ubiquitin to the phagophore, which itself is marked by the ubiquitin-like molecules LC3/GABARAP. Otherwise, organelle-specific receptors can also facilitate ubiquitin-independent autophagy [11].

When proteostasis is challenged, eukaryotic cells are capable of sensing and responding to proteotoxic insults through compartment-specific transcriptional programs, including the cytosolic heat-shock response (HSR) and the unfolded protein responses in the endoplasmic reticulum (ER) and mitochondria (UPR^ER^ and UPR^MT^), respectively [12–14]. A commonality of HSR and UPRs is the activation of key regulatory transcription factors (TFs), which occurs in a compartment-specific manner and triggers a dedicated compensatory response. The consequence of HSR/UPR induction is essentially to limit the load of unfolded and nonfunctional proteins by three means: (a) a decrease in global translation, accompanied with (b) increased expression of chaperones facilitating proteostatic capacity, and (c) enforced proteasomal degradation (Fig. 1). The integrated stress-response (ISR) is activated by various stressors, e.g., viral infection or Heme deprivation, but also in response to amino-acid shortage or ER-stress [15, 16]. Central to the ISR is the phosphorylation of eukaryotic translation initiation factor 2A (eIF2A), which triggers translational reprogramming through global inhibition of protein synthesis along with selective expression of particular stress-response genes harboring upstream open reading frames (uORFs). The physiological relevance of the described stress pathways is emphasized by various pathologies linked to impaired stress response and consequent loss of proteostasis [2, 7, 15].

### Metabolism and proteostasis in aging and disease

**Fig. 2:**
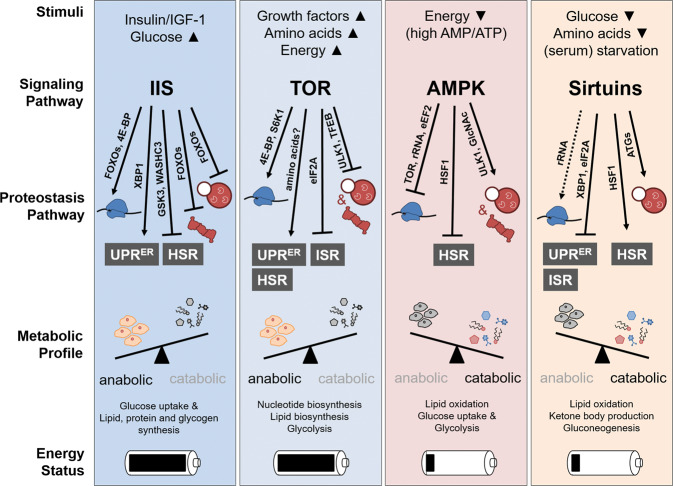
Metabolic regulation of proteostasis pathways. Schematic overview of central metabolic pathways. Arrowheads indicate the abundance of stimuli/metabolites activating respective signaling pathways (**▲** upwards, high abundance/**▼** downwards, low abundance). The effect of active signaling on proteostasis pathways is shown on the level of protein synthesis (blue), proteasomal and autophagosomal degradation (red), and responsiveness of stress-compensatory pathways (gray). The effect of pathway activation on the metabolic profile is indicated for anabolism (growth, proliferation) and catabolism (biomolecules, metabolites), respectively. The availability of cellular energy under physiological pathway activation is shown as loaded or empty battery icon, respectively. See text for details.

**Fig. 3:**
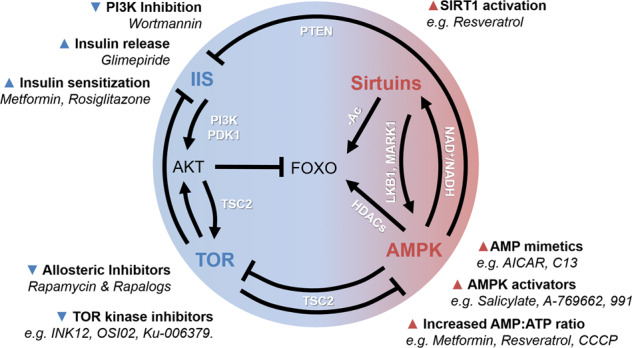
Orchestration of metabolic pathways. A schematic depiction of the interconnection between key metabolic pathways and pharmacological interventions. IIS and TOR signaling are activated upon energy availability and are connected via the serine/threonine kinase AKT. IIS promotes AKT activation in a PI3K- and PDK1-dependent manner. AKT in turn phosphorylates FOXO transcription factors and thereby prevents nuclear translocation. AKT and AMPK act antagonistically to regulate TOR signaling through inhibitory and activating phosphorylation of TSC2, respectively. AMPK and sirtuin activity are both induced by low-energy conditions. AMPK can possibly stimulate sirtuins by elevating the production the NAD^+^ biosynthetic enzyme Nampt, which increases the NAD^+^/NADH ratio. Vice versa, sitruins might deacetylate LKB1, which targets the AMPK-related kinase MARK1 ultimately enhancing AMPK phosphorylation. Sirtuin and AMPK signaling also promote FOXO-mediated transcriptional activity, either by direct deacetylation (−Ac) or by the phosphorylation-dependent activation of histone deacetylases (HDACs), respectively. Moreover, AMPK can block IIS by induction of the PIP_3_ phosphatase PTEN. Several pharmacological interventions are available that reduce (▼) or stimulate (▲) the activity of the different metabolic pathways and are in part used to treat metabolic disorders in human patients. See text for details.

Proteostasis mechanisms shape the proteome by regulating either the synthesis or degradation of proteins and are therefore connected to the cellular pool of amino acids that are utilized for protein biosynthesis or liberated during proteolysis. Both synthesis and degradation of proteins are processes that consume ATP and thus particularly conditioned by energy metabolism. Through a multitude of enzymatically catalyzed chemical reactions cellular metabolism essentially contributes to the production or turnover of cellular energy and structural biomolecules. Anabolic pathways are directed toward synthesis of higher-order biomolecules from small metabolites to generate biomass, critical for growth or as resource for energy-generating catabolic processes. Catabolic pathways use complex biomolecules, e.g., extracted from food, to turn them over into smaller metabolites that cells use to build macromolecules or generate energy. In healthy cells, anabolic and catabolic pathways are intricately orchestrated in order to achieve a balanced state of growth and energy homeostasis (Figs. 2, 3).

**Fig. 4:**
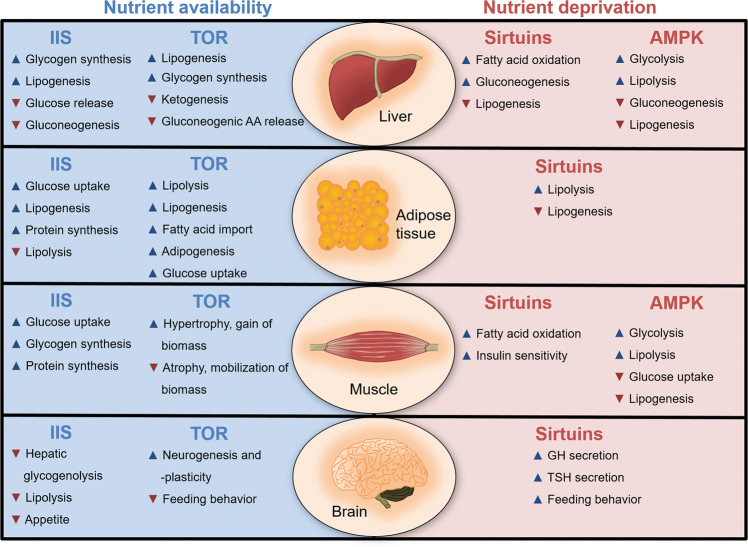
Organ-specific physiological regulation of metabolism. Overview on metabolic pathways and how they influence the metabolic profile of different tissues including, liver, adipose tissue, muscle, and brain. IIS and TOR signaling are activated upon nutrient deprivation and either trigger anabolic nutrient utilization or counteract catabolic processes that increase nutrient availability and lower appetite and feeding behavior. In contrast, AMPK signaling and sirtuins act in response to nutrient deprivation promoting nutrient replenishment but inhibiting nutrient/energy storage. Sirtuins promote feeding behavior and growth hormone (GH, somatotropin) as well as thyroid-stimulating hormone (TSH) secretion, which antagonize the action of insulin and increase fat breakdown to provide the energy necessary for tissue growth. Arrows indicate inhibition (**▼**) or stimulation (**▲**) of the metabolic processes.

A growing body of evidence points out that changes in metabolism signify a source of cellular stress [17]. In conditions of nutrient scarcity and consequently low ATP level, cells need to manage energy generation without undercutting overall cellular physiology [18]. Conversely, also overnutrition and excess metabolic activity can threat cellular function, e.g., through resulting metabolites or generation of reactive oxygen species (ROS). Increased energy generation and mitochondrial electron transport chain activity is a well-established source of toxic ROS level, which triggers oxidative damage of macromolecules and metabolic pathologies [19]. Next to ROS, other metabolites e.g., O-linked uridine-diphosphate-acteylglucosamine (UDP-O-GlcNAc) or Acetyl-CoA emerge as critical regulators of cellular physiology. UDP-O-GlcNAc originates from the carbohydrate metabolizing hexosamine pathway and serves as basis for posttranslational protein glycosylation. Acetyl-CoA is generated by Acetyl-CoA synthetase or catabolic cleavage of citrate and is used for acetylation of lysine-residues (K-acetylation). Noteworthy, O-GlcNAcylation and K-acetylation play critical roles in proteostasis maintenance by regulating gene expression, protein quality control and stress-response pathways as well as the UPS and autophagy [20, 21]. Coordination of metabolism and proteostasis is crucial for balanced energy homeostasis, organismal physiology and health (Fig. 4, Table 1). In contrast, deficits in proteostasis contribute to the development of metabolic diseases including diabetes and cancer [1, 2, 22]. Therefore, it is of outstanding interest to understand how metabolic perturbation and proteotoxicity are interconnected to design promising strategies for therapeutic interventions.

**Table 1:** Disease models linked to proteostasis decline are associated with altered metabolic activity.

Disease	Metabolic pathway	Effect on proteostasis	Organism	References
Neuro- and muscle degenerative disorders				
Alzheimer’s disease	IIS/DAF-2▼	Reduced aggregation-mediated Aβ toxicity	Ce	Cohen et al. [29]
	FOXO/DAF-16▲		Mm	Cohen et al. [165]
	Sirtuins▲	SIRT1 overexpression and resveratrol treatment reduced tau phosphorylation and Aβ accumulation/neurotoxicity	Rn	Chen et al. [166]
			Mm, Hs	Min et al. [167]
			Mm, Rn, Hs	Kim et al. [168]
	TOR▼	Activation of autophagy, clearance of Aβ-aggregates	Mm	Caccamo et al. [169]
Parkinson’s disease	Sirtuins▼	Deletion of Sir2 abrogates α-synuclein toxicity through modulation of autophagy	Sc	Sampaio-Marques et al. [170]
	Sirtuins▲	Resveratrol-mediated activation of SIRT1 enhanced α-synuclein degradation via autophagy induction	Hs, Rn	Wu et al. [171]
		Sir2.1 suppresses α-synuclein inclusion	Ce	van Ham [172]
	TOR▼	Activation of autophagy and the proteasome, clearance of α-synuclein aggregates	Rn	Webb et al. [173]
	TOR▼, AMPK▲	Activation of autophagy, clearance of preformed α-synuclein fibrils	Hs	Gao et al. [174]
	AMPK▼, constitutively active	Mitigation of α-synuclein toxicity	Mm	Bobela et al. [175]
	AMPK▲	PARP overexpression depleted ATP pool, suppressing neurodegeneration dependent on AMPK	Mm	Kim et al. [108]
Huntington’s disease	IIS▲ FOXO/DAF-16▼	More proteotoxic polyQ-aggregates in the background of reduced FOXO/DAF-16 signaling	Ce	Morley et al. [176]
				Hsu et al. [34]
			Mm	Cohen et al. [165]
	Sirtuins▲	Resveratrol supplementation protected against mutant huntingtin-induced cytotoxicity	Mm	Parker et al. [177]
	TOR▼	Activation of autophagy, clearance of polyQ-aggregates	Hs, Mm, Dm	Ravikumar et al. [178]
	TOR▼	Activation of autophagy, clearance of polyQ-aggregates	Hs	Roscic et al. [179]
	TOR▼	Perturbation of amino-acid levels inhibits TOR and activates autophagy	Dm	Vernizzi et al. [180]
	AMPK ▲	Reduced number of protein aggregates, induction of autophagy, improved neuronal function	Ce	Gómez-Escribano et al. [181]
Amyotropic lateral sclerosis	TOR▼	Mutations in *ubiquillin* genes induce ER-stress and proteotoxicity that reduces TOR signaling and induces autophagy	Dm	Şentürk et al. [85]
	TOR▼	Proteasomal degradation of VAB(P58S)-aggregates	Dm	Chaplot et al. [182]
	SOD1▼			
	Sirtuins▲	SIRT1 activation by resveratrol reduced SOD-1-mediated proteotoxicity	Mm	Markert et al. [183]
				Kim et al. [168]
Ischemic brain injury	TOR▼	Perturbation of amino-acid levels inhibits TOR and activates autophagy	Mm, Hs	Yamada et al. [184]
Prions disease	TOR▼	Decreased lateral spreading due to decrease in exosomal release	Mm	Abdulrahman et al. [185]
Duchenne muscular dystrophy	IIS▼ FOXO/DAF-16▲	Less protein aggregates and age-dependent proteostasis disruption in muscle cells	Ce	Oh and Kim [186]
Metabolic disorders				
Hyperinsulinemia/insulin resistance/deficiency	IIS▼	Increased (cardiac) muscle proteolysis promotes synthesis and stability of chaperones and UPR^ER^ components and suppresses autophagy	Mm, Rn	Wang et al. [49]
				Hu et al. [187]
				Yang et al. [188]
				Otoda et al. [189]
				Minrad et al. [190]
T1DM	IIS▼	Increased whole body protein degradation	Hs, Mm	Nair et al. [191]
		Increased proteasome function	Mm	Xu et al. [99]
T2DM	IIS▼	Increased protein aggregation in β-cells	Zr, Rat, Hs	Kaniuk et al. [192]
Hyperglycemia	IIS▼	Enhanced 26S proteasome functionality through peroxynitrite/superoxide-mediated PA700-dependent proteasomal activation	Mm, Hs	Liu et al. [193]
Cancer				
Glioblastoma	TOR▲	Gliomagenesis is promoted by a positive feedback loop involving TORC2/AKT/HSF1/RICTOR	Hs	Holmes et al. [194]
Multiple myeloma	AKT▼	Inhibition of translation initiation as target to induce breach of proteostasis and apoptosis in MM cells	Hs	Zismanov et al. [195]
	TOR▼			Zismanov et al. [196]
	Sirtuins▼	SIRT6 depletion renders cells tumorigenic. SIRT6 regulates cell proliferation by acting as a corepressor of c-Myc, inhibiting the expression of ribosomal genes	Mm	Sebastián et al. [197]

Table displays metabolic pathways with elevated (▲) or reduced (▼) activity and correlation to effects on proteostasis pathways in model systems of human disease. Model systems used in referenced studies are indicated as *Homo sapiens* (Hs), *Mus musculus* (Mm), *Rattus norwegicus* (Rn), *Zucker rat* (Zr), *Drosophila melanogaster* (Dm), *Caenorhabditis elegans* (Ce), *Saccharomyces cerevisiae* (Sc).

## Metabolic pathways that control proteostasis

Protein and metabolic homeostasis are two regulatory nodes for maintenance of cellular and organismal physiology. However, their functional interconnection and how perturbations in one system affect the other are widely underappreciated. In the following paragraphs we will provide an overview about the key metabolic pathways/regulators including insulin/IGF1 signaling (IIS), TOR, adenosine monophosphate-activated protein kinase (AMPK), and sirtuins and discuss the impact on proteostasis (Fig. 2). While this review article focusses on protein homeostasis, it should be noted that metabolic adaptations also occur in parallel to the regulations in proteostasis pathways, which is summarized in Fig. 4.

### Insulin/IGF1 signaling

As the prevalent hormone involved in the regulation of many important biological functions, insulin affects metabolism, cell growth, mitochondrial biogenesis, and physiology but also tissue differentiation and remodeling [23, 24] (Fig. 4). Consequently, perturbation of insulin/IGF1 signaling (IIS) is usually associated with metabolic diseases like Diabetes mellitus and obesity (Table 1). Insulin acts on many tissues with distinct effects on glucose, protein, and lipid metabolism (Fig. 4).

The IIS pathway is primarily activated by insulin and IGF1 (insulin-like growth factor 1). In the mammalian system, insulin/IGF1 binds to the IGF1/insulin receptor (IGF1R/IR) upon high blood glucose levels [24]. This class of tyrosine kinase receptors activates the insulin receptor substrate family which recruits the phosphoinositide 3-kinase (PI3K). PI3K activity leads to elevated tri-phosphorylated inositol PtdIns(3,4,5)P3 (PIP3) levels which activate the serine/threonine kinase AKT. Upon activation, AKT phosphorylates a variety of downstream effectors of IIS which further modulate glucose uptake by the glucose transporter type 4 (GLUT4), glycogen synthesis by glycogen synthase kinase 3 (GSK3), protein and lipid synthesis by TOR (mechanistic target of rapamycin, see below) and gene expression by forkhead box-O class (FOXO) TFs [25] (Fig. 3).

FOXO (DAF-16 in *Caenorhabditis elegans*) regulates the expression of target genes implicated in metabolism, cell growth, cell proliferation, stress resistance, and differentiation [26]. AKT-mediated phosphorylation of FOXO affects gene expression by nuclear exclusion of the transcription factor [27]. Loss-of-function mutations in foxo/*daf-16* negatively affect life span and stress resistance in flies, worms, rodents, and potentially also humans, indicating a central role in organismal longevity [23, 26]. In addition, decreased IIS has been shown to affect proteostasis during the aging process [28, 29] and the role of the IR homolog DAF-2 in organismal proteostasis and life span has been extensively studied in *C. elegans* [30, 31]. Although the underlying mechanisms supporting lifespan extension by reduced IIS are multilayered (Fig. 4), one of the major effects is through activation of FOXO and HSF1 (heat-shock factor 1), which positively affect proteostasis [32, 33]. HSF1 is a transcription factor central to stress responses induced by various stimuli not only including heat shock, but also osmotic or oxidative stress and glucose starvation. FOXO and HSF1 cooperate to regulate the transcription of target genes encoding for chaperones like small heat-shock proteins (HSPs) [34]. AKT phosphorylation also leads to the inactivation of GSK3, a negative regulator of HSF1 [35]. GSK3 has been implicated in a many human pathologies including cancer, neurodegenerative disorders and diabetes [36, 37]. GSK3 can phosphorylate HSF1 to inhibit its transcriptional activity by forcing its nuclear exclusion, thereby reducing expression of HSPs [38, 39]. This negative regulation of HSF1 implicates a novel role of GSK3 in proteostasis regulation and in some cancers reduced GSK3 activity may contribute to malignant transformation at least partially via HSF1 activation. In *C. elegans* IIS can negatively regulate HSF-1 through DDL-1 (homolog of human WASHC3), which binds to and represses HSF-1 [40]. Life span extension, mediated by decreased IIS, was shown to remodel the ER-stress response and to involve autophagy [41, 42]. TOR is activated by IIS, resulting in autophagy inhibition and increased protein synthesis [43]. Moreover, IIS activates XBP-1, a key component of the IRE1 sensor pathway of the UPR^ER^ via PI3K binding [44, 45].

The function of insulin in controlling glucose homeostasis is well established, whereas its role in maintaining proteostasis remained unclear. IIS controls protein synthesis/degradation and posttranslational modifications in different tissues, thus orchestrating proteostasis at the organismal level (Figs. 2, 4). Already early in vitro studies showed that insulin inhibits protein degradation in muscle [46], but further investigation of ubiquitin ligases and proteasome inhibitors provided insight into the underlying mechanisms of how insulin and IGF1 control protein degradation pathways. Studies in insulin-deficient rats revealed an increase in muscle proteolysis predominantly by ubiquitylation and proteasomal degradation which was suppressed by the proteasomal inhibitor MG132 [47, 48]. Similar effects have been observed in insulin-resistant, diabetic (db/db) mice [49]. In muscle, IGF1 and FOXOs are involved in the control of ubiquitin-mediated protein turnover (proteasomal degradation and autophagy) by regulating the expression of the E3-ubiquitin ligases atrogin-1 (MAFbx) and MuRF1 and autophagy-related genes [50–52]. A recent study suggests a direct impact of IIS on the modulation of proteasome activity via DAF-16-mediated downregulation of the proteasome-associated deubiquitinating enzyme (DUB) UBH-4 in *C. elegans*. UBH-4, also known as Uch37 in yeast, might act as a tissue-specific proteasome inhibitor and its function is conserved in mammalian cells, where downregulation of the UBH-4 ortholog UCHL5 leads to an increased degradation of proteotoxic proteins [53].

In *D. melanogaster* FOXO and its target Thor/4E-BP, a critical regulator of cap-dependent translation, have an important role in the pathogenesis of age-related muscle weakness. Increased activity of FOXO/4E-BP preserves muscle function by promoting autophagy which delays the age-associated decline in protein quality control. Moreover, FOXO/4E-BP signaling is involved in life span extension and regulates organismal proteostasis by modulating feeding behavior, insulin secretion, and 4E-BP induction in non-muscle tissues [54] (Fig. 4). Taken together these findings underline the direct impact of IIS on muscle-specific UPS regulation [51].

IIS integrates complex signaling events to maintain cellular homeostasis. Therefore, the functionality of IIS factors is tightly regulated by a variety of control pathways including the UPS [55]. On the one hand, the UPS shapes the activity of IIS via modulating the availability, stability, and activity of key regulatory factors through ubiquitin-mediated signaling [56]. On the other hand, insulin directly impacts the UPS by modulation of ATP production and gene expression [57]. Recent studies reported a complex crosstalk between IIS and UPS critical for maintaining proteostasis. In human skeletal muscle insulin triggers the expression of a number of E2 enzymes and proteasomal subunits [58] and also the DUB USP16, which further modulates gene expression through histone deubiquitylation [55, 58]. We have recently shown that the E3-ubiquitin ligase CHIP executes a dual function by either acting in chaperone-assisted degradation of misfolded and aggregated proteins or inducing endocytic-lysosomal degradation of the IR [33]. In this context, the overexpression of aggregation-prone polyglutamine (polyQ) shifted CHIP’s activity from IR binding to protein degradation in inclusion bodies, promoting IR stabilization, increased insulin signaling, and downregulation of longevity supporting genes, all of which are signs of aging. Detailed reviews about insulin signaling and its implication in proteostasis and metabolic disorders can be found elsewhere [59, 60] (Fig. 4, Table 1).

All together, these observations underline the fact that IIS and proteostasis mechanisms are part of a complex interwoven network of metabolic pathways. Thus, the dysregulation of insulin signaling caused by elevated stress conditions, aging, or other environmental insults can have major pathological consequences including neurodegeneration, muscle wasting, and diabetes (Table 1).

### TOR signaling

The mechanistic target of rapamycin (mTOR, or TOR) is the major regulator of anabolic pathways mediating growth under nutrient rich conditions [61]. TOR is the central serine/threonine kinase at the core of two structurally and functionally discrete signaling complexes, TORC1 and TORC2, respectively. TORC1 is a key regulator of cellular growth controlling protein biosynthesis, transcription, nutrient uptake, and energy expenditure as well as autophagic activity. Consistent with its central role in the regulation of energy homeostasis, TORC1 activity is closely coordinated with key metabolic pathways such as IIS, AKT, and AMPK (Fig. 3) as well as via endocrine signaling of the central nervous system. TORC1 is the direct pharmacological target of rapamycin, which inhibits TORC1 activity. The alternative TORC2 complex, however, has been shown to be inhibited only after prolonged rapamycin treatment [62]. TORC2 critically regulates cytoskeletal organization, and activity of several kinases (e.g., AKT and SGK1) and consequently associated downstream processes.

In contrast to IIS, which is responsive to high glucose conditions, TOR activity is regulated through amino-acid levels. Glucose levels, however, also impact on TOR indirectly through AKT, thus integrating IIS and stress-response signals (Fig. 3). TOR signaling attracted most attention due to its beneficial effect on aging and age-related diseases, either by pharmacological or genetic intervention. In several model organisms TOR inhibition increases life-expectancy and provides positive effects on metabolic parameters [63, 64], mitochondrial activity, and insulin sensitivity upon long-term rapamycin treatment [65]. Interestingly, lifespan extension by rapamycin in *Drosophila* was shown to be independent of impaired IIS or dietary restriction [66]. In fact, increased lifespan is mediated by TORC1-dependent regulation of proteostasis pathways including protein biosynthesis and autophagy [66].

Regulation of proteostasis via TOR signaling occurs on several levels, reshaping the cellular proteome (Fig. 2). Two central downstream effector proteins of TORC1, eukaryotic translation initiation factor 4E-binding protein (4E-BP) and ribosomal protein S6 kinase 1 (S6K1), control protein biosynthesis. Upon TORC1-dependent phosphorylation of 4E-BP, its sequestering activity towards eukaryotic translation initiation factor 4E (eIF4E) is released, consequently reinforcing translation initiation of 5′ capped mRNAs [67, 68]. Activation of 4E-BP upon TORC1 inhibition has beneficial consequences on several aging parameters and lifespan regulation [54, 69, 70]. Of note, downstream effectors of 4E-BP namely MTFP1, TFAM, and PGC-1-alpha stimulate health-promoting effects of 4E-BP via mitochondrial functionality [61, 63].

Similar to 4E-BP, S6K1 phosphorylation upon TORC1 activation promotes global protein biosynthesis [71, 72], whereas S6K1 inactivation increases lifespan and metabolic health [73]. Downstream targets of S6K1 again illustrate the intricate interconnection of signaling pathways linked to energy homeostasis. On the one hand, beneficial effects of S6K1 inactivation are genetically dependent on AMPK (see below) and likely do not result from global translation regulation [73]. On the other hand, S6K1 has been shown to impede IIS under nutrient rich conditions through inhibitory phosphorylation of IRS1 and IRS2 [69, 73].

In addition to its role in promoting protein biosynthesis, ubiquitin-mediated protein degradation via the proteasome and autophagy are regulated by TOR signaling. In contrast to enhanced protein biosynthesis, protein degradation is suppressed by TOR activation. Inhibition of both, TORC1 and TORC2 results in elevated ubiquitylation and protein degradation [74]. In both yeast and mammalian cells, TOR inhibition activates the Map-Kinases Mpk1 and ERK5, respectively, increasing the abundance of active proteasomes through chaperone-assisted assembly [75]. An independent study, however, reported decreased transcription of proteasomal subunits upon genetic TOR activation, which is dependent on the nuclear respiratory factor 1 [76]. These controversial observations on proteasome regulation by TOR might be explained by experimental context. Acute TOR inhibition by rapamycin treatment might promote proteasomal activity [74, 75], whereas chronic activation can also increase proteasome abundance, possibly to provide free amino acids for protein biosynthesis [76].

Aside from regulating proteasomal protein turnover, TOR is recognized as a well-established inhibitor of protein degradation by autophagy. Decreased autophagy is mediated through TORC1-dependent inhibition of UNC-51-like autophagy-activating kinase 1 (ULK1) [77] as well as inhibition of transcription factor EB (TFEB), preventing TFEB-mediated lysosomal biogenesis and autophagy [78–80]. Interestingly, also TORC2 activity has been linked to a selective form of autophagy that is mediated by chaperone-assisted lysosomal import. TORC2 that is localized to the lysosomal membrane inhibits import and turnover of chaperone-mediated autophagy substrates [81].

Of note, increased autophagic turnover has beneficial effects on aging parameters and cellular physiology and has been considered as health- and lifespan supporting mechanism of TOR inhibition. In this regard, the *let-7* microRNA was identified as a physiological regulator of autophagy in the context of TOR activation. Under nutrient starvation, *let-7* inhibits amino-acid sensing and consequently TOR activation to allow autophagy induction [82]. In the context of mechanical muscle stress, the co-chaperone BAG3 locally sequesters the TORC1 inhibitor TSC to facilitate autophagy induction at defined sites of filament damage, while at the same time allowing TORC1-mediated protein biosynthesis in the cytosol [83]. A recent study overexpressing molecular chaperones in yeast revealed that improved chaperoning capacity results in TORC1 inactivation along with AMPK activation, thus implying that proteostasis also serves as a regulator of metabolic pathways [84]. This concept is supported by findings in a *Drosophila* model of ALS. Loss-of-function mutation in ALS-linked ubiquillin genes, generates proteotoxicity and ER-stress, which in turn inhibits TORC1 activity and autophagy induction. In this case, however, autophagy induction is insufficient to compensate proteotoxicity due to failure in lysosomal acidification and consequent autophagic clearance [85].

Another proteostasis mechanism regulated by TOR signaling is linked to stress-response pathways of the ER. The two branches of the UPR^ER^, mediated through the ER-resident sensors IRE1 and PERK, are modulated by TORC1 activity. Whether TOR signaling also affects the third ATF6-dependend branch of the UPR^ER^ remains to be elucidated. Inhibition of TORC1 suppressed IRE1 and PERK signaling including downstream effector pathways such as JNK and NF-kappa-B [86–88]. A potential mechanism was suggested by the recent finding that lysosomal degradation and cytosolic resorption of amino acid is critical for ER functionality and prevents UPR^ER^ induction [89]. Besides UPR^ER^, PERK has another established role in ISR activation parallel to other activating kinases [15, 16]. TORC1 signaling impacts on eIF2A at multiple levels through either phosphorylation or de-phosphorylation steps. Activation of TORC1, via nutrient or growth factor supply or by genetic ablation of the TORC1 inhibitor TSC, results in eIF2A de-phosphorylation and thus inactivation of the ISR [88, 90]. Similarly, TORC1 inhibition results in elevated eIF2A phosphorylation which is necessary for efficient autophagy induction [91]. In the context of amino-acid deprivation the general control non-depressible protein 2 (GCN2) kinase activates the ISR through eIF2A phosphorylation [16]. Interestingly, GCN2 and TOR control protein synthesis in parallel pathways [92, 93], implicating GCN2 as another critical module connecting metabolic adaptation to proteostasis regulation.

### AMPK signaling

AMPK signaling acts as a pivotal sensor of cellular energy levels and becomes activated when available energy is scarce. AMPK activity is thus directed toward preservation of ATP through catabolic metabolism and simultaneous inhibition of anabolic pathways, including TOR signaling [94] (Figs. 3, 4). AMPK is activated by high AMP levels, allosterically [95] and indirectly through phosphorylation by liver kinase B1 (LKB1). Moreover, other molecules characteristic for cellular energy consumption activate AMPK [94]. Conversely, AMPK activity is inhibited when energy supply is high, e.g., through amino acids or AKT (Figs. 2, 3) and TNF-alpha signaling. The repertoire of substrates is huge and exemplifies the diversity of cellular processes regulated by AMPK [96]. In this regard it should be noted that AMPK constitutes a key regulator of cellular glucose and lipid catabolism, aiming at ATP generation (Fig. 4). Consequently, dysregulated AMPK signaling has been intensively linked to metabolic pathologies including diabetes, cancer, cardiovascular disease, neurodegeneration, and aging (Table 1).

A connection between AMPK signaling and proteostasis is well established at the level of proteasomal protein degradation (Fig. 2). For instance, high levels of extracellular glucose are known to increase the activity of the 26S proteasome. Of note, increased proteasomal activity under high glucose levels is dependent on AMPK activity. Both pharmacological activation or expression of a constitutively active AMPK variant suppressed glucose-induced proteasome activity [97]. Accordingly, genetic ablation or inhibition of AMPK leads to enhanced proteasomal activity [97, 98]. The molecular mechanism of proteasome inhibition by AMPK remains less defined. One study reports a role of posttranslational modification of the 19S regulatory particle of the proteasome by O-GlcNAcylation. O-GlcNAcylation of proteasome subunits suppresses the assembly of the 26S holoenzyme and consequently its degradation capacity [20]. Inhibition of AMPK activity is linked to concomitant increase in 19S O-GlcNAcylation, thus explaining increased 26S proteasomal activity [99]. An alternative regulatory mechanism was proposed based on the protein–protein interaction of AMPK with the proteasomal subunit PSMD11 (Rpn6p in yeast) [100]. How phosphorylation of PSMD11 is correlated with proteasomal activity remains to be determined. In this regard it should be mentioned that PSMD11 has been recognized as a potent and evolutionary conserved determinant of proteasomal activity ensuring stress resistance and longevity [101, 102]. As elevated proteasomal activity upon PSMD11 expression is dependent on the FOXO4 transcription factor, regulation of proteasome activity by AMPK and IIS signaling might be cross-connected via controlling PSMD11. Next to regulation of the proteasome, AMPK signaling has also been implicated in controlling the activity of several ubiquitin E3 ligases, which could also impact cellular proteostasis [103]. Pharmacologic activation of AMPK has been shown to increase protein degradation in muscle cells through increased expression of atrophy-related ubiquitin ligases, depending on the FOXO1 and FOXO3 TFs [104]. Protein turnover in muscle tissue is attributed to the UPS upon exercise. In a human study of trained athletes, exercise-induced proteolysis could be partially suppressed by administration of branched-chain amino acids, which was correlated with elevated AMPK activity [105], suggesting a physiological role of AMPK in controlling proteostasis in muscle. Experimental activation of AMPK through elevated AMP/ATP ratio revealed the transcription factor HSF1 as a direct target of AMPK phosphorylation, diminishing HSR induction, consequently rendering cells sensitized to proteotoxic stress [106]. This finding is physiologically relevant as proteotoxic stress inhibits AMPK activity, allowing adaptive transcriptional response via HSF1 [106] (Fig. 2).

In the context of proteostasis mechanisms critical in lifespan regulation, AMPK signaling has been linked to the autophagic degradation as well. Under nutrient scarcity AMPK activation drives the restoration of cellular amino-acid levels and probably other metabolites through lysosomal turnover and subsequent resorption into the cytosol (Fig. 2). Autophagy regulation by AMPK is mediated through cAMP-regulated transcriptional coactivators (CRTCs), modulating cAMP-responsive element binding protein (CREB) signaling [107]. Direct substrates of AMPK are components of the autophagy regulating complexes TORC1, ULK1, and PIK3C3/VPS34 that contribute to autophagy induction [77, 108]. In addition, AMPK controls autophagy in an indirect manner through the transcriptional regulation of target gene expression of the TFs FOXO3 [109], TFEB [110], and BRD4 [111]. Alternative to autophagosome formation and subsequent lysosomal fusion, degradation inside the lysosome is also mediated through direct vesicular uptake from the cytosol, called microautophagy. In yeast cells starved for glucose, compromised proteasome particles are selectively removed via microautophagy in an AMPK-dependent manner. This regulation allows storage of functional proteasomes in cytosolic deposits, which facilitates rapid resumption of proteasomal activity upon glucose availability [112]. In addition to the inhibitory role of protein quality control and degradation, AMPK attenuates protein biosynthesis at several steps. In parts, AMPK inhibits protein biosynthesis by TOR inhibition [113, 114] but also by TOR-independent negative regulation of ribosomal RNA (rRNA) synthesis [115] as well as translational elongation [116, 117].

It should be noted that AMPK activity, aside from its impact on proteostasis that is depicted here, is a nexus for the regulation of diverse metabolic processes which are intricately interconnected (Figs. 3, 4). AMPK emerges as a critical regulator of mitochondrial biogenesis through PGC-1-alpha and NAD-dependent acetylation, which has strong implications in protein quality control mechanisms [118, 119].

### Sirtuin signaling

Sirtuins are evolutionally conserved members of the atypical class III histone deacetylase family, which require nicotinamide adenine dinucleotide (NAD) as cofactor [120]. As an important metabolic molecule NAD directly links the activity of sirtuins to the metabolic state of the cell. Sirtuins sense energy states by detecting high NAD levels. Seven mammalian sirtuins, SIRT1–7, have been identified to regulate a variety of biological functions including stress responses, cell cycle regulation and metabolism [121]. In the nucleus, SIRT1, SIRT6, and SIRT7 epigenetically shape gene expression by deacetylating histones [122]. SIRT2 is considered cytosolic, but has also been described to modulate cell cycle progression in the nucleus [123]. The mitochondrial SIRT3, SIRT4, and SIRT5 regulate metabolic enzyme activity and oxidative stress [124] and respond to caloric restriction (CR) by inducing cellular programs that favor mitochondrial oxidative metabolism as wells as increasing stress tolerance. CR upregulates SIRT1, SIRT3, and SIRT5 in mice and SIRT1 in humans [122]. In contrast, mice fed a high-fat diet show a loss of SIRT1 via proteolysis [125] and obesity can lower the expression of SIRT1 in humans [126]. Upon fasting or CR, SIRT1 contributes to coordinating a metabolic switch from anabolic to catabolic processes, including glycogen degradation and subsequent gluconeogenesis as well as ketone body production, lipolysis, and lipid oxidation to maintain energy levels [127] (Fig. 4). In this context, SIRT1 deacetylates and regulates the activity of metabolic enzymes and TFs [128].

The effects of sirtuins on life span an tumorgenesis have been heavily debated in the past years. Depending on the molecular context and cancer type, some sirtuins function as proto-oncogenes, while others exhibit features of tumor suppressors. Despite recent reports that challenge the positive effects of sirtuins on lifespan extension [129], it has been shown that nutrient limitation supports longevity sirtuin-dependently [130] and overexpression of sirtuins extends life span [121, 131, 132]. Moreover, the fact that sirtuins require NAD for their enzymatic activity directly links metabolism to aging and aging-associated diseases. A comprehensive overview on the controversial discussion about the impact of sirtuins on lifespan an tumorgenesis can be found elsewhere [133].

Among other longevity pathways, sirtuins interact with FOXO TFs to target cellular pathways (Fig. 3), that are often deregulated during aging such as metabolism, cell cycle progression and cellular senescence as well as stress resistance and programmed cell death [134, 135]. Sirtuins deacetylate FOXO TFs in response to cellular stress which positively affects lifespan in worms, flies and mammals [23, 133, 136].

So far, the impact of sirtuins on aging is mainly linked towards CR, regulation of energy metabolism (Fig. 4), and control of circadian rhythm as well as cell death [122, 133–135]. However, a potential role of sirtuins in maintaining cellular proteostasis introduces a new perspective for sirtuin-mediated lifespan regulation (Fig. 2). NAD-dependent life span extension in aged worms and mice requires sirtuins to activate both, FOXO/DAF-16-mediated antioxidant stress signaling and the UPR^MT^ [137]. A crosstalk between sirtuins and UPR^ER^ has been reported in mammals, whereby SIRT1 deacetylates the IRE1‐dependent transcription factor XBP-1 to inhibit its activity [138, 139]. SIRT1 also suppresses translational inhibition by PERK‐eIF2A [140]. Moreover, SIRT7 positively affects translation by promoting the transcription of ribosomal RNA by RNA polymerase I, thereby contributing to ribosome biogenesis [141] (Fig. 2).

Furthermore, SIRT1 might support proteostasis and protect against proteotoxicity through activating HSF1. In mammalian cells, SIRT1 directly modulates the HSR by deacetylating HSF1, suggesting that the HSR is under metabolic control. Along that line, a recent study in *C. elegans* uncovered a *sir2.1-*dependent synergistic effect of CR and HSR on the induction of *hsp70* gene expression, thereby increasing survival and fitness upon proteotoxic stress conditions [142]. Furthermore, it was reported that the SIRT1 homolog in yeast, Sir2, interconnects the UPR^ER^ and HSR [143], which is necessary for HSR induction and that Sir2 overexpression mimics HSR [143, 144]. The authors demonstrated that activation of the UPR^ER^ by tunicamycin treatment upregulates Sir2, which acts as a negative modulator of the UPR^ER^ ensuring only transient activation of this pathway. Sir2 and an intact UPR^ER^ are required for eliciting HSF1-mediated HSR, which impacts on stress-response networks amongst different organelles [143].

As described above, CR and nutrient scarcity modulate nutrient-sensing and metabolic pathways, including sirtuins, TOR, and AMPK, to promote longevity possibly by regulating autophagy [145, 146]. In contrast to the TOR pathway, Sir2 activates autophagy in yeast [147]. In mammalian cells, SIRT1 plays an important role in maintaining autophagy upon nutrient deprivation by regulating the autophagic factors ATG5, ATG7, ATG8, and ATG12 via deacetylation [148]. For a more detailed overview, the role of sirtuins in autophagy has been recently reviewed [149]. Interestingly CR and sirtuins and their influence on autophagy have been linked to the pathogenesis of age-related neurodegenerative diseases including AD, HD, PD, and ALS (Table 1).

These observations collectively show that besides energy metabolism (Fig. 4), sirtuins also impact on proteostasis mechanisms and cellular stress-response pathways. Especially the ability to regulate autophagy renders sirtuins as promising therapeutic targets for fighting neurodegenerative disorders.

## Discussion

This review provides an overview of how key metabolic regulators impact on proteostasis mechanisms. Both, metabolic imbalance and proteostatic decline are causally linked to aging and aging-associated pathologic disorders (Table 1), underscoring the need to identify common signaling nodes and shared mechanisms. Despite numerous implications for an intricate interconnection of both processes, metabolism and proteostasis are mostly studied from independent perspectives. Future research is needed to address the impact of declined proteostasis on metabolic diseases and whether targeting protein quality control mechanisms might be a relevant clinical intervention. Conversely, targeting metabolic signaling pathways should be considered as possible strategy for treatment of neurodegenerative disorders.

One interesting question remains how viability and functionality are ensured when turning on/off growth pathways that remarkably remodel the cellular proteome. This implies that specificity in protein biosynthetic and degradation pathways need to exist. In the case of selected regulation of stress-response genes through uORFs or 5′-capped mRNAs, global translation inhibition can be coordinated with simultaneous activation of tailored adaptive responses. Conversely, little is known about the selectivity of protein degradation pathways in that context. In this regard, two recent comprehensive proteomic studies suggest that autophagic degradation upon TOR inhibition or amino-acid withdrawal specifically affect autophagic degradation of ER components (ER-phagy) but not ribosomes (Ribo-phagy) to regenerate amino-acid levels [150, 151]. Proteomic analyses in long-lived *C. elegans* mutants lacking the insulin receptor DAF-2 revealed a significant increase in global protein half-life. However, it remains to be examined how a long-lived proteome contributes to organismal health and longevity and what are the molecular mechanisms that preserve proteostasis under these conditions [152, 153]. Generally, enhanced autophagic degradation has established beneficial effects in health parameters and models of neurodegenerative diseases (Table 1). In contrast, the contribution of translational regulation to proteostasis maintenance in the context of metabolic regulation remains to be further explored.

The disease-related accumulation of neurotoxic aggregates and misfolded proteins is often accompanied with systemic changes in metabolism occurring also in distal tissues lacking protein aggregates. Likewise, stress responses often trigger cell-non-autonomous communication which allows organism-wide regulation of proteostasis [154–156]. However, the mechanisms by which the central nervous system induces a response towards proteotoxic stress in distal tissues remained unknown. Although there is clear evidence that proteostasis networks are regulated in a cell-non-autonomous manner there are still many open questions on how the different layers of proteostasis control are coordinated and which factors mediate the interorgan communication. External stimuli play a key role in the context of organismal regulation of proteostasis and metabolism. Amongst them, a balanced diet, food quality, and microbiota composition define stress resistance and organismal health. Besides cognitive decline, a common pathology in neurodegenerative diseases include gastrointestinal symptoms and dysfunctions [157, 158]. Recent research focused on deciphering the underlying communication pathways between gut bacteria and the central nervous system (microbiota–gut–brain axis) and dysregulation in this communication have been implicated in the pathophysiology of neurological diseases [159]. Moreover, a diverse, healthy diet correlates with diverging gut microbiota, which is associated with improved overall global indices of health, frailty, and immune function, especially in the elderly population [160]. However, not only the food intake and the composition of the microbiome but also food perception has an influence on metabolism. Recent studies implicate that sensory perception modulates energy homeostasis via integration of neuronal signals originating from sensory tissues [161, 162]. Moreover, olfactory dysfunction has been acknowledged as a symptom present in dementias and the elderly population [163]. Interestingly, our recent study links sensory food perception to organismal proteostasis in *C. elegans*, which is cell-non-autonomously communicated via neuronal signaling between olfactory neurons and intestinal cells [164]. However, it is still unclear how environmental and/or nutritional cues are incorporated into proteostasis pathways and how this could be used as health benefit. Irrespective of the progress made in characterizing systemic stress-response programs, a major challenge in this research area is to extend our fundamental understanding of the mechanistic coordination of energy metabolism, microbiota–gut–brain axis, food perception and the control of protein quality mechanisms.
