# Correction to: Build-UPS and break-downs: metabolism impacts on proteostasis and aging

**DOI:** 10.1038/s41418-021-00800-4

**Published:** 2021-05-11

**Authors:** Franziska Ottens, André Franz, Thorsten Hoppe

**Affiliations:** 1 Institute for Genetics and Cologne Excellence Cluster on Cellular Stress Responses in Aging-Associated Diseases (CECAD), University of Cologne, Cologne, Germany; 2 Center for Molecular Medicine Cologne (CMMC), University of Cologne, Cologne, Germany

**Keywords:** Protein quality control, Metabolic pathways, Ageing

Correction to: *Cell Death & Differentiation*

10.1038/s41418-020-00682-y

The article Build-UPS and break-downs: metabolism impacts on proteostasis and aging, written by Franziska Ottens, André Franz, Thorsten Hoppe, was originally published electronically on the publisher’s internet portal on 4 January 2021 without open access. With the author(s)’ decision to opt for Open Choice the copyright of the article changed on 12 January 2021 to © The Author(s) 2021 and the article is forthwith distributed under a Creative Commons Attribution 4.0 International License, which permits use, sharing, adaptation, distribution and reproduction in any medium or format, as long as you give appropriate credit to the original author(s) and the source, provide a link to the Creative Commons licence, and indicate if changes were made.

The images or other third party material in this article are included in the article’s Creative Commons licence, unless indicated otherwise in a credit line to the material. If material is not included in the article’s Creative Commons licence and your intended use is not permitted by statutory regulation or exceeds the permitted use, you will need to obtain permission directly from the copyright holder. To view a copy of this licence, visit http://creativecommons.org/licenses/by/4.0/. In addition, figure 8 was printed in b/w although it was submitted in color. Open Access funding enabled and organized by Projekt DEAL. This has been corrected in the original article.

